# Re “International survey on skin patch test procedures, attitudes and interpretation” L.K. Tanno et al., WAOJ (2016) 9:8

**DOI:** 10.1186/s40413-017-0149-0

**Published:** 2017-04-26

**Authors:** Wolfgang Uter, Magnus Bruze, Thomas Rustemeyer, David Orton, Vera Mahler, for the European Society of Contact Dermatitis

**Affiliations:** 1 Department of Medical Informatics, Biometry and Epidemiology, University of Erlangen/Nürnberg, Waldstr. 6, 91054 Erlangen, Germany; 2 Department of Occupational and Environmental Dermatology, Skåne University Hospital, Lund University, 205 02 Malmö, Sweden; 3 Department of Dermatology, VU University Medical Centre, 1081 HV Amsterdam, The Netherlands; 4 Hillingdon Hospital, Uxbridge, UB8 3NN UK; 5 Allergy Unit, Department of Dermatology, University Hospital Erlangen, 91054 Erlangen, Germany

**Keywords:** Patch testing, Standardization, Survey

## Abstract

A previous survey on the practice of diagnostic patch testing among representatives of member societies of the World Allergy Organization (WAO) has, in some countries, not addressed those stakeholders actually involved in patch testing, mainly dermatologists. The need for further standardisation is acknowledged and has been addressed e.g. by publication of a patch test guideline by the European Society of Contact Dermatitis in October 2015.

Dear Editor,

We read with interest the survey results presented by L.K. Tanno et al. [1] conducted among the representatives of member societies and members of the Junior Members Group of the World Allergy Organization (WAO) late 2013/early 2014. Reviewing the member societies in Europe (http://www.worldallergy.org/about-wao/member-societies, last accessed 5 April 2017) we noted that regarding some countries, there seemed to be a mismatch between the societies approached, and those societies and medical specialities, respectively, who actually perform patch testing in a given country. As just one example, in Germany patch testing is almost exclusively performed by Dermatologists [as is the case in some other (European) countries], but the respective dermatology subspecialty group [in this case, the German Contact Dermatitis Research Group (http://dkg.ivdk.org/, last accessed 5 April 2017)] was not approached—which is probably why Germany remained *terra incognita* on the world map of respondents, together with Sweden, Finland and a number of other countries who lead in contact allergy research. Moreover, none of the international networks active in the field of contact dermatitis and patch testing were approached, such as the American Contact Dermatitis Society (www.contactderm.org) and the North American Contact Dermatitis Research Group, respectively, or the European Society of Contact Dermatitis (www.escd.org).

Notwithstanding the bias introduced by a failure to approach relevant scientific societies across different regions of the world, we fully agree with the authors about the need for standardisation—as in every medical diagnostic and therapeutic procedure. There seems to be an apparent lack of awareness of pertinent guidelines. In order to improve this situation we would like to take the opportunity to point out the patch testing guideline elaborated by the European Society of Contact Dermatitis (ESCD) published in 2015 [2]. This is available with “free access” from the publisher’s website (http://onlinelibrary.wiley.com/doi/10.1111/cod.12432/full, last accessed 5 April 2017), which should further promote awareness and, hopefully, adherence. The society’s journal “Contact Dermatitis” (2015 IF: 5.7) is dedicated to best practice. We also would like to point to the fact that the ESCD is a relatively small, but very active society in the field of contact dermatitis and patch testing, with members from many different countries and all continents. Young researchers in particular who are interested in this field, including standardisation aspects, are warmly welcomed to join in (see https://www.escd.org/membership/, last accessed 5 April 2017).

We consider it essential that a patch test service is run by a physician with relevant training in dermatology—ideally a Dermatologist (see [2], Supplemental information S1). However, we do acknowledge that in some countries patients with dermatitis may be seen initially or in entirety by Allergists. Whenever Allergists are taking care of children or adults with chronic or recurrent dermatitis, including atopic eczema, they must be able to provide their patients with necessary patch testing, preferably by referral to, or at least collaboration with, a Dermatologist who regularly performs patch testing. If this is not feasible, then patch testing can be done by the Allergist, in order not to compromise access to this important diagnostic tool, and not to delay diagnosis of contact allergy. Nevertheless it is essential for any clinician performing this investigation to have the necessary knowledge, proper training and experience to achieve the best outcome for the patient.

## Abbreviations

ESCD: European Society of Contact Dermatitis
WAO: World Allergy Organization
